# Prioritizing rehabilitation in low- and middle-income country national health systems: a qualitative thematic synthesis and development of a policy framework

**DOI:** 10.1186/s12939-023-01896-5

**Published:** 2023-05-17

**Authors:** Rachel Neill, Yusra Ribhi Shawar, Lamisa Ashraf, Priyanka Das, Sarah N. Champagne, Hunied Kautsar, Nukhba Zia, Georgia J. Michlig, Abdulgafoor M. Bachani

**Affiliations:** 1 Johns Hopkins International Injury Research Unit, Health Systems Program, Department of International Health, Johns Hopkins Bloomberg School of Public Health, 615 N. Wolfe Street Suite E8527, Baltimore, MD 21205 USA; 2 Department of International Health, Johns Hopkins University Blomberg School of Public Health, Baltimore, MD USA; 3 Paul H. Nitze School of Advanced International Studies, Johns Hopkins University, Washington, DC USA

**Keywords:** Rehabilitation, Health policy, Governance, Health priorities, Low- and middle-income countries, Global health, Politics, Disability

## Abstract

**Background:**

There is a large and growing unmet need for rehabilitation – a diverse category of services that aim to improve functioning across the life course – particularly in low- and middle-income countries. Yet despite urgent calls to increase political commitment, many low- and middle-income country governments have dedicated little attention to expanding rehabilitation services. Existing policy scholarship explains how and why health issues reach the policy agenda and offers applicable evidence to advance access to physical, medical, psychosocial, and other types of rehabilitation services. Drawing from this scholarship and empirical data on rehabilitation, this paper proposes a policy framework to understand national-level prioritization of rehabilitation in low- and middle-income countries.

**Methods:**

We conducted key informant interviews with rehabilitation stakeholders in 47 countries, complemented by a purposeful review of peer-reviewed and gray literature to achieve thematic saturation. We analyzed the data abductively using a thematic synthesis methodology. Rehabilitation-specific findings were triangulated with policy theory and empirical case studies on the prioritization of other health issues to develop the framework.

**Results:**

The novel policy framework includes three components which shape the prioritization of rehabilitation on low- and middle-income countries’ national government’s health agendas. First, rehabilitation lacks a consistent problem definition, undermining the development of consensus-driven solutions which could advance the issue on policy agendas. Second, governance arrangements are fragmented within and across government ministries, between the government and its citizens, and across national and transnational actors engaged in rehabilitation service provision. Third, national legacies – particularly from civil conflict – and weaknesses in the existing health system influences both rehabilitation needs and implementation feasibility.

**Conclusions:**

This framework can support stakeholders in identifying the key components impeding prioritization for rehabilitation across different national contexts. This is a crucial step for ultimately better advancing the issue on national policy agendas and improving equity in access to rehabilitation services.

## Background

There is a growing unmet need for rehabilitation – a category of interventions aimed at improving functioning and reducing disability [1]. Rehabilitation services are important across people’s life span and include physiotherapy, occupational therapy, speech and language therapy, audiology, post-injury or post-surgical care, the provision of assistive technology and devices, and psychosocial services including psychotherapy and counseling [1].

One in three persons may require rehabilitation services within their lifetime [2]; however, access to those needed services remains inequitable. Less than 50% of individuals requiring rehabilitation in low- and middle-income countries (LMICs) can access the care they need, limiting quality of life and increasing disability [1]. Persons with a disability are more likely to experience poverty, face additional barriers in accessing health services, and can experience social inequalities [3].

The critical importance of rehabilitation in improving quality of life has led to its inclusion in international policy frameworks, including the Declaration of Alma Alta [4] and the United Nations Convention on the Rights of Persons with Disabilities [5]. The World Health Organization’s (WHO) recent Rehabilitation 2030 Initiative seeks to elevate rehabilitation as an essential health service and a core component of Universal Health Coverage (UHC) and advocates for, “strong leadership and political support for rehabilitation at sub-national, national and global levels” [6].

But despite increasing global recognition, rehabilitation receives little attention from most governments in LMICs, with even fewer nations implementing adequate policy to support the advancement of rehabilitation services in national health systems [2, 7–9]. This raises a key question: why is rehabilitation rarely prioritized?

Theories from the social sciences explain how issues are elevated to the policy agenda and can help to understand the prioritization of rehabilitation in LMICs [10]. Agenda-setting theory has identified that policymakers do not automatically focus on issues with the most evidence; instead, they direct their limited resources towards specific areas identified as a problem and seen as amenable to intervention [11]. A leading agenda-setting theory is Kingdon’s multiple stream theory [12]. Kingdon argues that there are three streams – problem (transformation of a societal issue into a specific problem), policy (actors who advance policy solutions), and politics (macro-level changes such as political representation, national mood, or protests) – that come together to create a window of opportunity when advocates can advance an issue onto the policy agenda [12].

Kingdon’s multiple streams and broader policy scholarship emphasizes key factors that influence prioritization. These include the extent to which there is consensuses on the nature of the problem and its solutions (problem definition) [12–15], the way in which actors organize to advance collective action (governance) [16–18], and the role of institutions or structural factors in constraining available choices [11, 16, 19].

Health policy scholarship applies agenda-setting theory to understand how and why specific health issues rise on national or global agendas. The Shiffman and Smith framework on determinants of political priority for global health initiatives is a commonly utilized framework for health issue prioritization [16]. Originally developed from case studies on maternal mortality, it delineates a range of factors known to influence the prioritization of health issues at the global level [16]. These include actor power (strength and cohesion of actors concerned with an issue), ideas (understanding and communication of an issue), global political contexts, and issue characteristics (such as indicators, severity, and available interventions) [16]. Existing health policy case studies illustrate the utility of agenda-setting theory and the Shiffman and Smith framework in understanding health issue prioritization [17, 20–32]. They also demonstrate considerable variation in the specific factors that drive prioritization for different health issues [17, 20–32].

We identified no previous research on rehabilitation’s prioritization on national or sub-national health agendas in LMICs. A 2016 review on the governance of rehabilitation found little evidence on agenda-setting factors and called for additional research [33].

### Objective of this study

The objectives of this study are to (1) identify the key factors that shape rehabilitation’s prioritization across LMIC national health systems, and (2) to distill these into a novel policy framework to guide future research and practice. In this paper, we analyze key informant interview data, complemented by a purposeful selection of published and gray literature, to identify the key factors that shape prioritization of rehabilitation. Guided by existing health policy scholarship and agenda-setting theory, we then synthesize these factors into a policy framework specific to the prioritization of rehabilitation in LMIC national health systems. The framework provides a path forward for rehabilitation stakeholders to better understand and address the critical factors that can advance prioritization of rehabilitation, a key first step to improving equitable access to rehabilitative services.

## Methods

Following a commonly utilized definition in the health policy process literature developed by Shiffman (2007), prioritization is defined in this study as concern for the issue, the enactment of policies that advance consensus-based solutions, and the consistent application of public funds aligning with the unmet need [20]. This definition encompasses both political attention – high level attention, targets, and coordination – and political commitment – the allocation of resources, accountability, authority, and oversight to policy formulation [21].

We use two sources of data to understand the prioritization of rehabilitation in national LMIC health systems. Our primary data source was qualitative key informant interviews (KIIs) with transnational and national rehabilitation stakeholders. We complemented these interviews with our second data source, which was a purposeful review of peer-reviewed and gray literature on governance and policy for rehabilitation. The KIIs were the foundation of our analysis and were the key data source used to develop the framework. The rehabilitation-specific literature aided in the initial coding of the interview data by highlighting potentially applicable themes across a range of countries and contexts, providing additional theoretical and empirical evidence to support interview themes, and providing more country case examples to further enrich the interview data.

We complemented the KII data and rehabilitation-specific peer-reviewed and gray literatures with theories from the agenda setting phase of the policy process, which informed our analysis. A thematic synthesis methodology was used to abductively analyze both the KIIs and the rehabilitation-specific literature against the policy theory to develop the policy framework. Thematic synthesis is utilized to integrate findings of qualitative research and is appropriate for the generation of interpretive constructs [34]. We reported our processes via the Standards for Reporting Qualitative Research [35].

### Identification of relevant agenda-setting theory and health issue prioritization

Sociological theory directs us to the interaction between agentic and structural factors that shape the policy prioritization process [36, 37]. Agentic factors are the capacity of actors to act independently within structures – in other words, their agency [36, 37]. In the policy process, agents compete to exert their issue on a limited agenda.

To deepen our understanding of how health issues are prioritized and apply these insights to rehabilitation, we reviewed scholarship from public administration and policy on governance, advocacy, and the agenda setting phase of the policy process [12, 18–20, 38–42], and empirical case studies on the prioritization of other health issues including maternal mortality, rheumatic heart disease, violence against children, emergency care, global surgery, early childhood development, nutrition, global disease control, newborn survival, drowning, and pneumonia [15–17, 20–32]. These takeaways informed our initial data extraction approach and supported the iterative assembling and reassembling of the data described further in the analysis section.

### Identification of secondary data on governance and policy for rehabilitation

Following the recommendations of thematic synthesis [34], we completed a targeted hand-search of both peer-reviewed and gray literature to identify relevant secondary data on governance and policy for rehabilitation. We searched ten, peer-reviewed literature databases – PubMed, Embase, CINAHL, PsycINFO, Scopus, Web of Science, PAIS Index, JBI Database of Systematic Reviews and Implementation Reports, 3iE, and WHO Global Index Medicus – and the Google search engine. We began our search in the academic databases by conducting a search for relevant keywords in the title or abstract. Search words related to ‘rehabilitation or functioning’ and ‘policy, health service delivery, health systems, governance, economy, institutions, and politics’ were used.

We reviewed titles and abstracts for relevance in the order they were provided by the search engine, downloading the full text of a paper if the title or abstract appeared relevant, and then reviewed the full text to determine relevance of the paper against pre-determined inclusion and exclusion criteria (Table 1). The inclusion and exclusion criteria were developed based on a group discussion within the research team, all of whom have prior experience working in rehabilitation, health policy, or health systems. Because of our focus on agenda setting, we focused our search broadly on ‘upstream’, or macro-level political, economic, and bureaucratic, factors influencing prioritization and excluded papers that were focusing solely on micro- or meso-level factors without any link to macro-level factors. We excluded vocational rehabilitation because vocational rehabilitation is often within the mandate of labor laws and government agencies and therefore, we saw this as potentially a distinct category as it relates to prioritization. Similarly, we expected the factors related to substance abuse prioritization to be specific to the needs of a specific population. Finally, anticipating that the literature specific to rehabilitation in LMICs would be limited, we allowed for the inclusion of multi-country studies that included high-income countries in our purposeful search.

**Table 1:** Inclusion and exclusion criteria for peer-reviewed and gray literatures

**Criteria**	**Inclusion**	**Exclusion**
Definition of rehabilitation	• Aligned with the World Health Organization’s definition of rehabilitation [1], including the rehabilitation of conditions arising from injury, surgery or post-surgical care, disease, mental health, congenital conditions, age, or illness	• Vocational rehabilitation • Rehabilitation services specific to substance abuse needs
Scope	• The political, economic, and bureaucratic factors that impact health policy prioritization of rehabilitation and/or assistive technology • The prioritization and implementation of policies that advance rehabilitation and/or assistive technology in national health systems	• Only includes meso- or micro-level factors – for example, articles with an exclusive focus on service delivery, program evaluations, or articles describing target group or provider experiences with rehabilitation programs
Publication data	• Published after 2000, since the momentum around rehabilitation and its integration into health policy and systems research was gained at the turn of the century including its inclusion into global goals like universal health coverage	• Published prior to 2000
Level of focus	• At the country or global level • If a multi-country paper, any country income level • If a single country case study, focused on a low- or middle-income country	• Papers focused exclusively on a single, high-income country case study
Language of publication	• English language publications	• Publications written in a language other than English

When a relevant paper was identified per the inclusion and exclusion criteria, an initial data extraction was completed. This allowed us to build an early conceptual understanding of the sampled literature and informed our iterative and non-exhaustive search process.

We continued searching for and reviewing identified literature until we reached a point of conceptual saturation, where additional papers were adding little to no new information to our initial data extraction [34], and when we repeatedly identified the same citations via snowball sampling from reference lists and in subsequent academic databases and the Google Scholar search engine.

### Key informant interview data

A purposive, maximum variation sampling approach was utilized to sample key informants with extensive research and/or practice experience in rehabilitation and health systems. KIIs with 65 health systems and rehabilitation stakeholders, representing 47 countries across all WHO regions and all country income levels, were conducted via Zoom from February 2020 and April 2021.

We purposefully sampled key informants (KIs) based on their perceived ability to contribute key insights on rehabilitation and the health system, including governance, policy, and leadership and based on access via the research team’s professional networks. Guided by the principle of maximum variation, we also sought to include KIs working in different professional capacities, including national LMIC government officials, rehabilitation health care providers, health professional associations, academic researchers, representatives from non-governmental or civil society organizations, leaders of disability peoples’ organizations, and international intergovernmental organizations (Table 2). We included at least one informant from each WHO region.

**Table 2:** Key Informant Characteristics

**Informant Profile**	**Total (n)**
National, LMIC government officials	13
Rehabilitation health care provider / sector	10
Health professional organization	7
Academia or research	13
Non-governmental or civil society organizations	10
Disabled People’s Organization	2
International intergovernmental organization	10
**Total (n)**	**65**

Sampled participants were contacted via email and invited to participate. KIs with a non-response or refusal were not included. All KIs had extensive professional experience working in rehabilitation and health systems, either at the national level in an LMIC or at the global level in an international organization or research institute.

Data was iteratively reviewed during the data collection process, and we stopped contacting new participants when we reached a point of saturation – in other words, gaining little new insights during new interviews. We considered all interviews equally when conducting the analysis, so as not to preference any one perspective in our analysis.

A semi-structured interview approach was utilized. KIs were asked about their definition of and perspectives on rehabilitation, leadership, governance, policy, and political factors shaping rehabilitation, rehabilitation’s role in the health system, and how to strengthen rehabilitation in their context. Interviews were conducted in English and Spanish by three interviewers, all of whom had experience working in rehabilitation in health systems and formal training in qualitative research methods. Interviews were assigned to the interviewer based on language; all interviewers in English were done by a single researcher, and a second researcher joined the first interview conducted. All interviews in Spanish were conducted by a different researcher who was a native Spanish speaker. English recordings were recorded in full, and the recording was transcribed verbatim. Spanish recordings were translated into English and transcribed in full. Participants were assigned an interview number in chronological order of the completion of the interview to maintain anonymity.

The study was deemed exempted, non-human subjects research by the Institutional Review Board of the author’s institution. Oral consent was confirmed prior to conducting each interview.

### Analysis

We took an iterative, abductive approach to data analysis, following the steps of thematic synthesis [34]. Themes were identified inductively and iteratively compared to policy theories in a process of categorizing and recategorizing the data into the final framework categories, with memos produced at each stage and an audit trial maintained [43, 44]. This is detailed in Table 3.

**Table 3:** Process of constructing the framework

	**Step**	**Description of the analysis process**
1	Line-by-line extraction of secondary data	• Included literature was read in full and data was extracted into Microsoft Excel • The following categories were used for the initial extraction: paper objective, stakeholders mentioned, summary of structural and agentic features influencing prioritization and implementation of rehabilitation policies, and reflections of the data extraction team
2	Development of descriptive themes	• An abductive analytical approach was utilized, moving between the extracted literature data and policy theory to distill themes under structure and agentic features
3	Generation of analytical themes	• A second round of thematic analysis was used to inductively identify analytical sub-themes from extracted literature within the structural and agentic categories • Group discussions within the research team were held to review sub-themes and consider representativeness across the literature • All extracted literature was re-assembled by sub-theme
4	Dualist inductive/deductive coding of key informant interviews	• Pre-coded segments of transcripts from the key informant interviews were re-coded against the themes and sub-themes identified from the extracted literature • A dualistic technique of deductive/inductive thematic analysis was utilized, comparing the extracted literature data and key informant interview findings. Additional sub-themes were added inductively based on the KII data
5	Triangulation with policy theory and framework finalization	• Equal weighting was applied to all forms of data when constructing the framework; however, the majority of the empirical data was derived from the KIIs • Abductive analysis was utilized to analyze how the final set of inductively generated categories emergent in the rehabilitation-specific data aligned with policy theory and scholarship

For example, literature data emphasized that rehabilitation lacks a common understanding across stakeholder groups. This finding was initially extracted as an ‘agentic feature’ — any factor largely within an actor’s power to control or act upon— influencing rehabilitation in step one. In step two, a review of empirical cases and policy theory emphasized the importance of ‘problem definition’ to prioritization, which aligned with the inductively identified challenge of ‘lack of common understanding of rehabilitation’. In step three, we reassembled the extracted data into a new theme labeled ‘problem definition’, which captured findings related to how rehabilitation was (or was not) understood and influencing factors therein. In step four, the problem definition code was utilized deductively to code KII transcripts. The interview data aligned with literature findings by helping us contextualize the initial finding through national-level examples of definitions as shared by KIs. Finally, in step five, both KIIs and the extracted data from the rehabilitation-specific literature data coded as ‘problem definition’ were analyzed against the broader policy theory and evidence from the prioritization of other health issues to consider how lack of a common definition of rehabilitation may influence prioritization in this case.

## Results

This study analyzed 65 KIIs and 56 peer-reviewed or gray literature documents, representing insights from over 50 countries, regional insights from sub-Saharan Africa, Latin America, and the Caribbean, and generalized literature on LMIC experiences.

We identified three components— problem definition, governance, and structural factors —shaping the prioritization of rehabilitation in national health systems. These are defined in Table 4.

**Table 4:** Components shaping the prioritization of rehabilitation in national health systems

**Components**	**Sub-components**	**Description**
**Problem definition**	Problem clarity	Common understanding of the definition and nature of rehabilitation
	Solution acceptability^a^	Ability to reach consensus on solutions to advance rehabilitation policy and services, which are perceived as politically feasible and acceptable in the domestic context
**Governance**	Domestic advocacy coalitions	The cohesiveness, representativeness, and power of domestic proponents working to advance rehabilitation on the national agenda, inclusive of government and non-governmental actors
	Transnational actors	The engagement of non-domestic actors concerned with rehabilitation – including donors, international organizations, and non-governmental organizations– through policy frameworks, normative influences, funding, and technical assistance
**Structural factors**	National legacies	The political and historical contexts that structure decision making and the existing rehabilitation system
	Health system structures	The arrangements of health services, their financing, and processes for data collection and reporting

^a^ In this usage, solutions are considered a part of an issue’s problem definition because solutions carry implicit assumptions about what a challenge, or problem, means and the feasibility of addressing it through policy intervention

**Fig. 1:**
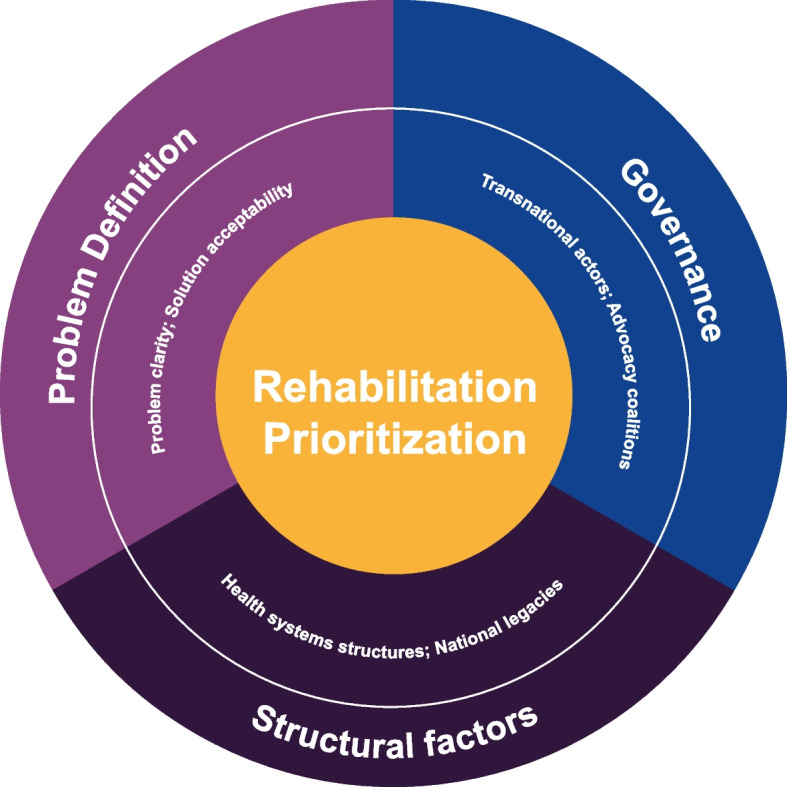
Framework for the prioritization of rehabilitation

These components are interconnected with prioritization at the center, as illustrated in Fig. 1.

Inconsistencies and complexity in the problem definition suggests internal framing contestation on rehabilitation, with lack of evidence on proposed solutions. Varied understanding of the problem results in fragmented domestic coalitions with some influence from transnational actors. These actors operate within historical legacies and existing health system structures which further shape how the problem is understood, the solutions that are advanced, and the actors involved.

Below, we present a comprehensive synthesis of our finding against each component of the policy framework for rehabilitation.

### Problem definition

Existing theory instructs that an issue is more likely to advance if it has a unified problem statement [12, 13], if the issue is seen as a “socially credible threat” [14], and if stakeholders advance agreed-upon solutions. Without consensus, the perception of ‘intractability’ may dissuade policymaker action [13].

We identified two problem definition components influencing the prioritization of rehabilitation: 1) clarity on the nature of the problem and 2) agreement on required and acceptable solutions. Lack of consensus in these two areas challenges prioritization on national policy agendas.

#### Problem clarity

In problem clarity, barriers to prioritization include 1) a lack of common understanding of rehabilitation across stakeholder groups [45, 46] and 2) challenges in making the case for rehabilitation as an urgent societal problem.

 Related to the lack of a common understanding, rehabilitation is defined and communicated in many ways – including as a health service, development imperative, human rights concern, and substance abuse issue [46]. The WHO has advanced an inclusive definition of rehabilitation focused on functioning across the life course [1]. ‘Improved functioning’ has united rehabilitation actors; however, there are competing internal definitions, or frames, within the rehabilitation community on whether improved functioning requires biomedical or psychosocial interventions. Both perspectives frame rehabilitation around functioning and both use the terminology of disability, but the implicit boundaries of what constitutes disability and functioning are different.

In the biomedical definition, rehabilitation is considered within the boundaries of the health service delivery system, focused on disability resulting from a traumatic or non-traumatic cause. The definition focuses on body borne conditions, including stroke, congenital conditions, injuries, and orthopedic conditions (S021, S022, S026, S054, S017, S018, S005, S020, S029, S047, S049, S054, S058, S047, S050) [47]. Often, the problem definition is linked to the epidemiological transition including the growing burden of noncommunicable diseases, aging, and increase in traffic-related crashes and injuries. An informant describes this:

*“We have a huge burden of Chronic Disease, […] so that’s one huge problem, physical Rehabilitation. The second is the extent of injuries in our country. Also elderly. Certainly among those who’s had some incident, a stroke, an injury, or a fracture or something, to prevent contractures and get back to normal.”*— S054

From the biomedical perspective, understanding rehabilitation in relation to persons with disabilities (PWD) could attach stigmatizing views to the sector and/or exclude other populations in need of support [8, 46, 48, 49]. Many informants utilize the term disability, but the implicit definition of disability is linked to a specific, body borne condition or disease which is amendable to medical intervention. For example:

*“[W]e have survivors with disability, a cancer survivor, a cardiovascular survivor, etcetera, etcetera, so we will have more survivors with disability.”* – S058

In contrast to the biomedical perspective, a broader, psychosocial approach to rehabilitation includes how enabling or impeding factors from outside a person’s body influence their functioning (S054, S011, S034, S010, S040, S049, S017, S023, S039) [50, 51]. This social and relationally-oriented framing emphasizes social and environmental factors including the engagement of communities, broader social and attitudinal factors (such as stigma), environmental structures (such as the presence or absence of physical accessibility), and intersectional vulnerabilities. These informants did not exclude health services but described their definition as more holistic than the biomedical model:

*“[Rehabilitation is] a really holistic process. […] The aim is to rehabilitate or bring back a function that has been lost somehow and depending on what are the areas, you will rehabilitate the body, or you will rehabilitate the society or you will find tools in order to include the person in the society*.” – S017

Informants that articulated a psychosocial definition also used the term disability in relation to rehabilitation. However, there are differences in the breadth of disability and how the term is being utilized. The key difference is the inclusion of social and environmental factors:

*“Until you have people reporting on spheres of disadvantage, […] you’re not going address disability, and it’s going to remain a kind of token gesture. [..] It needs to be more looking at sort of social, political and economic determinants of health”* – S010

The extent to which informants articulate personal, environmental, and social factors as it relates to disability also varied. One informant described this by using the term impairment versus disability:

*“One, what is impairment, and second, what is a disability. […] Impairment is an organic problem, and the disability is a social problem. Society is the one that imagines a person with some deficiency and speaks of them as disabled than deficient*.” – S024

Finally, a critique among a small number of informants describing a pyscosocial definition was that rehabilitation could be misconstrued with the idea that someone with a disability needs to be rehabilitated to achieve a ‘normal’ state (S046, S052, S008) [48].

In failing to present a common definition, policy makers and the broader population misunderstand rehabilitation and corresponding policy needs [45, 46]. It was suggested that Ministry officials had similar gaps in understanding as the broader population (P045, P026, S016, S060) [45, 52–54]. In the words of one informant:

*“Even a Ministry of Health… [doesn’t] realize what real rehabilitation means. […] He thinks it is sanatorium.”* –S060

Lack of clarity on the nature of the problem can impede stakeholder’s ability to convince others that the problem demonstrates a “socially credible threat” requiring prioritization. Rehabilitation stakeholders face challenges articulating loss of functioning or disability as a population health threat. Policymakers respond more urgently to threats of mortality, particularly from infectious diseases, compared to morbidity (S024, S025, S008, S030, S047, S040, S001) [55, 56]. One informant shared their experience making the case for rehabilitation compared to infectious disease:

*“I was working in the Ministry, in the National Office of Rehabilitation [and…] we presented a project that did not proceed. All because of diarrhea, we had polio and some other diseases that affect children under one-year-old. Infectious disease is one of the biggest obstacles we have.”* – S024

Improved functioning is a prospective benefit to individuals and society; however, problems are seen as more ‘threatening’ when they entail a loss, rather than a future benefit or a change in the current status quo [25]. This tension was articulated by advocates for rehabilitation who described an emphasis on preventing mortality (a loss) without consideration for reducing morbidity (a benefit).

“*Millions, millions [of dollars] in preventing malnutrition, prevention of diarrhea, but what is not taken into account is that malnourished children are going to need physical rehabilitation as well.*”. – S024

This may have resulted in a limited awareness of how rehabilitation could improve functioning further (S002, S007, S009, S018, S039, S049, S054) [52]. One informant described this dynamic:

*“[The] people’s general perception, particularly the ones who are not so educated, is that it’s okay, once you’ve had a fracture, you know it heals and you’re fine. The notion of making sure rehabilitation is done to bring it back to its original [functioning] is really not that widespread”. – S054*

These dynamics could result in rehabilitation being perceived as a ‘luxury’ rather than necessity [45, 57], further challenging prioritization.

#### Solution acceptability

Proposed solutions to strengthen rehabilitation in LMICs are often complex and unproven, which can dissuade prioritization. Most respondents argued for the integration of rehabilitation services into the health system, which aligns with existing global recommendations [7], but KIs articulated divergent views on how rehabilitation service should be integrated.

There was a tension between management of rehabilitation through a horizontal approach (integrated into primary health care (PHC) and other service packages) versus vertical approach (starting with a rehabilitation-specific package or hospital care). Starting with hospital-based care and strengthening referrals acknowledges current limitations with delivering rehabilitation in PHC (P003, P004, S004, S005, S006). One informant shared:

*“Primary care is missing on rehab. I am not aware of any primary care facility in India, which has rehab facilities. So again, that is a huge gap – my point would be not even to look at primary care” – P003*

An opposing approach was to start with rehabilitation’s integration into PHC (S025, S031, S026, S008, S014, S021, S024, S029, S030, S031, S035, S036). An informant described this approach:

*“[W]e integrate the rehabilitation programme, at health extension programme [PHC] level, that’s great. The second one is […] a health centre […] near to the deep rural community. […] These two groups, the large majority of the population […] can access.” – S002*

Another option was to integrate rehabilitation through specific disease entry points (S009, S058, S003, S002, S014, S021, S025). One informant described this solution:

**“***We don’t need another international plan of rehabilitation; we need to integrate rehabilitation in all parts of the health conditions…When we talk about international strategy of management of [non-communicable disease] NCD, we should integrate rehabilitation.”* – S058

Despite divergence in the proposed solutions, nearly all informants identified limited evidence for integration into health systems as a key challenge (S025, S036, S005, S025, S029, S031) [58–61]. Some argued for a long-term vision for what integration of rehabilitation means in practical terms (S031, S014, S009, S032). An informant articulated this:

*“We need to say in the next 10 years this is how I see rehabilitation services are integrated with good access throughout the continuum of care and this is how I'm going to start in a very implemental way, and this is how I'm going to evaluate as I move forward, and this is how I'm going to finance it.” – S*031

A possible enabler to generate solutions is international influence. Literature and interviews emphasized the importance of WHO guidelines and legislation from other countries to advance solutions [60, 62, 63]. In particular, the current leadership from WHO on rehabilitation and UHC serves as a rationale for promoting rehabilitation’s integration into the health system. The WHO’s Systematic Assessment of Rehabilitation Situation (STARS) health systems assessment tool, for example, offers a standardized approach to assessing capacities and setting rehabilitation priorities for national and sub-national agendas which could support consensus-based solutions [64].

### Governance

Effective governance is critical for generating collective action for advancing an issue [16]. Fragmentation, lack of role clarity, and competitive incentive structures impede prioritization, particularly for health areas that are multisectoral or horizontally organized [22, 23, 25, 65].

Domestic advocacy coalitions are often important to advancing prioritization [20, 26, 27]; however, there is tension between specificity versus diversity. Successful coalitions require a common identity but broad enough goals to include actors with political power that can increase advancement of the rehabilitation agenda [15, 27, 29, 31, 42]. Transnational actors can raise attention and lend normative pressure to an issue through the introduction of external resources, high-level policy frameworks, agreements, and measurement tools [17, 29]. However, their involvement may impede ownership, exacerbate fragmentation, or result in resource competition [32, 33, 35, 37].

Rehabilitation’s governance is hampered by lack of a common problem definition and complicated by the multisectoral nature of rehabilitation. Both governmental and non-governmental actors influence via domestic advocacy coalitions, while transnational actors exert influence on rehabilitation’s prioritization via funding and advocacy.

#### Domestic advocacy coalitions

Across our findings, there was a perceived need for increased advocacy and awareness raising of rehabilitation compared to other issues. One informant described the urgency of positioning rehabilitation at the policy level:

“*If you don’t develop strategy, [then] you don’t do work with other countries and provide information for policy makers to make [them] aware of the problem*." – S005

In maximizing the influence of domestic coalitions, theory points to the importance of leveraging political windows when coalitions can influence the policy process [12]. Political windows for advocacy and awareness raising were identified, including incorporating rehabilitation into existing primary health care reforms (S054), UHC initiatives (S045), and emphasizing the potential increase in rehabilitation needs as an aftermath of the COVID-19 pandemic (S025, S032, S036).

For government actors, KIs frequently suggested that political champions are needed within government ministries (P003, S002, S004, S021, S025, S045, S006, S015, S038, S027, P004, S037, S015, S058, S008, S017, S048, S013). One informant argued:

*“We don’t have individuals who are really driving it very hard. That’s what is needed. Once you have people take it up with the government, with the agencies, with the ministries, you get it taken care of because it's a problem that everybody is aware of.”* – S013

But where do government champions come from, and do they have similar interests? Adopting a problem definition beyond a biomedical perspective makes rehabilitation a multisectoral issue, and we found evidence for this within existing governance arrangements. In many countries, policies, funding, and programming for rehabilitation are found across Ministries of Health (MOH), as well as social welfare, education, labor, transportation, and justice [10, 45, 49, 62].

There is often no guiding institution within government to coordinate proposals or champion the issue, which may challenge collective action. Informants and literature described a lack of intersectoral coordination across ministries, including unclear or overlapping programs, accountability structures, and poor communication (S001, S002, S004 S027) [55, 66–70]. Fragmentation is further hampered by competing interests across agencies [9, 46, 49, 55, 62, 66–68, 71]. In the words of one informant:

**“***There is the Ministry [of Health]. Then, there are other institutions, like the national social security system […] along with another area called the Superintendency of Occupational Risks. […] there are many institutions at work […] and they cannot agree on anything”* – S027

The most common recommendation from KIs on how to improve governance was generating support from MOHs (P003, S002, S004, S021, S025, S045, S006, S015, S038, S027, P004, S037, S015, S021, S058, S008). Institutionalization of rehabilitation within a MOH has been recommended to strengthen governance, and Chile illustrates this potential. A strategic alliance between the Mental Health Unit, which had technical expertise, and the Primary Health Care Division, which had more power and resources, was critical to establishing guiding leadership [72]. With the MOH as the guiding institution, the Ministry of Women also came on board to support scale-up of depression treatment [72].

 In addition to the emphasis on government champions, national non-governmental organizations (NGOs) or civil society organizations (CSOs) participate in the policy process (S001, S008 S045 S004 S012, S029, S058). The strength of domestic advocacy coalitions is context specific. Some informants argued for further capacity (S026, S045, S058) [47, 60], while others emphasized the ability of coalitions to exert political pressure [57, 73, 74].

The ability of coalitions to influence decision-makers is partially dependent on their cohesion. In general, our data illustrated that health actors emphasize a biomedical framing while CSOs draw from a rights-based framing, which could impact the ability of coalitions to advance a common agenda.

In the biomedical framing, the emphasis is on the burden of disease and technical capacities for service delivery. This constituency is likely to align themselves with MOH. The role of provider associations was considered important in elevating rehabilitation within the health agenda (P004, S021, S017, S032, S039, S007) [46, 67]. However, a potentially limiting factor to health professional advocacy is the extent to which they have a collective identity as ‘rehabilitation’ providers (P004, S017, S026, S002, S034). One informant explained:

*“Professional organizations, first of [all], do not have an awareness that they work for rehabilitation. The physical therapist work[s] and advocate[s] for physical therapy; the occupational therapist for occupational therapy…”* – P004

In contrast, CSOs are often focused on PWDs, seen to promote a rights-based approach (P004, S033), and connect national-level work to international treaties [56, 74, 75]. PWD CSOs may or may not be engaged in advancing rehabilitation within the health sector. In Ghana, for example, a national disability consortium group was not engaged with the ‘medical rehabilitation’ system and efforts to advance training on rehabilitation [76]. Conversely in Uganda, disability rights organizations have been engaged in efforts to advance community-based rehabilitation programmes; however, these are governed under the Ministry of Gender, Labour and Social Development [77].

An additional complication is that representation of disability and rehabilitation within a single advocacy movement could be challenging. PWD movements themselves are not always representative. In the words of one informant:

**“***I think people with physical disabilities who are probably better able to self-advocate tend to command the large majority of resources…so the ability to advocate for resources and access resources is dominated by people who are physically disabled, [as opposed to] people with cognitive or mental illness…”* – S010

#### Transnational actors

Tensions exist surrounding the influence of transnational actors – including governments and other donor agencies providing international development assistance and/or supporting external health expenditures, global health initiatives, international agencies, and international NGOs – in advancing rehabilitation on national agendas.

National CSOs and NGOs were seen as connected to donor agencies providing technical and financial support, which was perceived to amplify their impact (S008, S048, P004, S046, S014, S029) [60, 78]. However, many national actors’ are dependent on external financing for their operations. This both threatens their sustainability and links their agendas to external donor priorities and funding (S004, S030, S048, S054, S040) [68, 73, 78, 79]. While some informants saw external financing of rehabilitation programs as a motivator for government action (S030, S004), a larger number of informants felt that there was little global interest or consensus on funding rehabilitation compared to infectious diseases such as HIV, Tuberculosis, malaria, and other infectious diseases (S002, S024, S039, S025, S029, P002) [55]. An informant describes:

*“There was the Millennium Development Goals […] and for 15 years, everybody focused on them […] but there was no rehabilitation component or aspects of such a disease programs. […] You wouldn’t expect countries to give priority to rehab if, at a global level, there is no shared consensus or shared understanding that rehabilitation should be a major aspect of the programs”* – S028

Specific to international organizations, interview data highlighted that the WHO is a leading convening power [70] and suggested an opportunity for countries to align with Rehabilitation 2030, a global initiative to raise awareness of the importance of rehabilitation [46]. KIs also encouraged the WHO to keep the pressure on governments:

*“You have the 2030 rehabilitation but like you know what I don’t know what else can be driving force for that? That could be or like a couple of champion countries regions that you know during the World Health Assembly […] are getting up and making statements in support or pushing for this rehabilitation to constantly be on the agenda”* – S029

An example of transnational advocacy for a rehabilitative service comes from the Pan American Health Organization (PAHO). In Latin America and the Caribbean, PAHO has raised the profile of mental health services through alignment with and knowledge sharing on WHO mental health policies and programs, including knowledge sharing initiative across countries, the facilitation of specific policy initiatives, and provision of technical assistance [60]. This support resulted in PAHO’s Directing Council of Ministries of Health to adopt a strategic plan on mental health [60].

This transnational advocacy has to be carefully translated to the national context, however. KIs at the national level described how international examples or advocacy can be perceived negatively if not suited to the specific needs of the country, dissuading prioritization by national policy makers (S040, S050, S048, S10, S027, S040). An informant explains:

*“[International organizations need to] dedicate the time to study the local manifestations that each population has. [gives ex from depression] I think that the WHO tries to do it, but to truly promote that, the WHO officials themselves or the people who [do the] work, do not stop traveling to the countryside to be close to the populations.” – S040*

Finally, despite considering WHO the main international champion of rehabilitation, it is possible that issue gatekeepers within the WHO have not adopted rehabilitation as widely as other health issues. This was alluded to by informants who pointed to the lack of inclusion of rehabilitation in global disease guidelines, which they felt directly influences the issue’s low prioritization at the national level:

*“WHO make[s] lots of guidelines, for example management of cancer, and they don’t talk about rehabilitation as part of the management of cancer. They make lots of guide[lines] [for] NCD, and they don’t talk about the [importance] of rehabilitation.”* – S058

### Structural factors

The first two components of our framework consider the agency of actors to advance policy change. In contrast, structural factors are the ‘rules of the game’ that constrain or enable the ability of actors to advance an issue [19, 65]. For example, political systems vary on their levels of participation and how power and influence is exerted in the policy process [19]. Historical contexts influence how issues are understood and framed which in turn influences prioritization [16, 80]. Health system structures may influence prioritization by exerting path dependency in the policy process [19]. Finally, resource constraints, both human and financial, are also structural factors that actors must contend with.

In the case of rehabilitation, national legacies and health system capacity are important structural factors shaping the issue’s policy advancement.

#### National legacies

National legacies impact an issue’s framing, perceived urgency of the problem, and service delivery arrangements. Most frequently, past or current conflict was linked to increases in disability, increased need for specific types of rehabilitation services, and the influx of foreign financial resources and NGOs. Conflict shaped prioritization of specific types of rehabilitative services and drove investments in specific areas.

In Kenya, political conflict led to an increase of funding to NGOs for mental health work [78]. In Morocco, an informant explained how an earthquake in 1960 and later armed conflict led to increase in rehabilitation services; however, the growth was confined to military hospitals (S005). In Guatemala, the legacy of guerrilla warfare impacted rehabilitation in the military medical system and launched broader social rehabilitation efforts to reintegrate soldiers into society (S033). In Vietnam, the legacy of civil conflict led to visibilities of PWD, and government funding allocated to support persons disabled due to the war, which has shaped the problem definition of rehabilitation towards physical disabilities (S021). In Colombia, the influence of civil conflict was also linked to an increased awareness of physical rehabilitation and improved assistive technology (S017).

Similarly, the 2015 conflict in Eastern Ukraine resulted in an increased burden of injuries and disability, drawing attention to the lack of health system capacity to treat those impacted by conflict and leading to new rehabilitation professional training programmes to build workforce capacity [51]. However, these efforts were seen as still limited by the Soviet legacy of hierarchical care and association of disability with ‘being an invalid’ [51, 70]. Finally, both Angola and Mozambique adopted community-based rehabilitation programs to provide rehabilitative services to conflict-impacted populations [81].

Other national legacies influencing rehabilitation include natural disasters (S029) [75], the role of devolution in changing institutional arrangements and accountability [78], the legacy of colonialism in mental health provision (S057) [60], elections [72], and legacies of the Soviet system (P004) [70, 82]. These can positively or negatively impact prioritization. Advancing the agenda, the 2015 earthquake in Nepal led to an increased burden of disabilities and spurred the creation of a Disability and Rehabilitation Unit within the MOH and a new ‘Policy, Strategy, and Ten Years Action Plan on Disability Management' [75, 83]. In Mexico, an informant shared how the polio epidemic led to training of rehabilitation professionals:

*“More widely since the polio epidemic in 1950, rehabilitation services were important, […] Specialists began to be trained, already with university knowledge. From that moment on, there started to be doctors specializing in rehabilitation.”* – S038

In contrast, economic turmoil in Zimbabwe led to an increase in mental health conditions while also reducing available financial and human resources to meet increasing need [55]. These examples illustrate the importance of understanding national legacies as a component of prioritization.

A cross-cutting national legacy is the role of stigma in influencing how rehabilitation is framed and therefore, its relative importance to both policy makers and the general population. Cultural perceptions of disability, attitudinal barriers, discrimination from family members and communities, and a general dismissive attitude were highlighted by KIs (S043, S010, S026, S005, S024, S033) as well as in the literature [47, 51, 52, 66, 69, 75, 76, 84]. Government officials and health providers were seen to hold similar stigmatizing views as broader society (P003, S017) [69]. This impacts prioritization, as policy makers often prioritize benefits for populations that are more powerful or are perceived as ‘deserving’, which can then further reinforce the construct that certain populations should not be prioritized [85].

#### Health system structures

We identified three health systems challenges influencing prioritization which are related to rehabilitation’s historic lack of representation in the public health care delivery system.

First, weaknesses in existing service provision included the lack of available or trained rehabilitation service providers, particularly in the public sector (P003, S054, S018, S008, S003, S014, S004, S005, S009, S015, S017, S021, S057, S054, S026) [51–53, 55, 58–60, 63, 68, 69, 78, 83, 86, 87], lack of appropriate infrastructure especially for in-patient services (S058, S026) [9, 51–53, 58, 60, 78, 83, 86, 87], gaps in supplies and assistive technology procurement (S046, P004, S015, S002) [83], and challenges in accessing essential medicines for mental health [55, 58, 68, 78, 86]. Lack of existing capacity to provide rehabilitation services may contribute to the perception of intractability and challenge the development of consensus-based solutions.

Second, private providers (including for-profit and not-for-profit, both formal and informal, and those receiving external financing) provide a large percentage of rehabilitative care in LMICs. This is closely linked to the role of transnational actors. Many not-for-profit service providers are funded by international development agencies and/or private philanthropy, raising concerns about sustainability and fragmentation (S023, S048) [66, 68, 78, 79, 81]. An informant describes the risks of externally-financed, private service provision:

*“[NGO service provider] would set up a building called community mental health service […].Three years, four years down the line, the fund comes to an end, everyone goes home”* – S033

Some KIs expressed concern that the private sector’s large role in care provision had displaced government interest and results in further fragmentation of a unified vision for rehabilitation services (S027, S024, S025). An informant described this dynamic:

*“The private entities are doing the work that the State should be doing. […] So, the State and the Ministry of Health are not interested, because other entities are taking charge of partially solving the problem.”* – S024

Third, there is a lack of national-level data on rehabilitation, which is related to how data systems are structured [88, 89]. This contributes to challenges in determining disease burden and unmet need for rehabilitation care, as well as efforts to illustrate improved outcomes due to rehabilitation services. This is crucial data for policymakers to understand the severity of the problem and its tractability and to monitor interventions and their impact. KIs emphasized the need for stronger evidence to make the case for rehabilitation, including cost-effectiveness, improved productivity, and improved health outcomes for other conditions (P004, S058, S004, S005, S007, S031, S054, S055).

## Discussion

Based on an analysis of KIIs and a complementary review of literature, we identified three interrelated components – problem definition, governance, and structural factors – impacting the prioritization of rehabilitation in LMIC national health systems. Within these components, we draw attention to how rehabilitation is understood (requiring biomedical or broader societal interventions), the relative ability of both government and non-governmental actors at the national and transnational levels to work collectively, and national legacies and health systems structures that shape the decisions of rehabilitation actors. These components have been distilled into a novel policy framework for the prioritization of rehabilitation in national LMIC health systems. This framework can be used to analyze the factors influencing prioritization of rehabilitation in different national contexts and to formulate context-specific strategies for advancing access to rehabilitation services.

Despite diversity across national contexts, national rehabilitation stakeholders face similar types of challenges in increasing the prioritization of rehabilitation on national agendas. Through application of the framework and triangulation with policy theory and evidence, we advance three interrelated considerations for policymakers and the rehabilitation community.

### Implications for the prioritization of rehabilitation

Divergent problem definitions challenge prioritization. A high degree of homogeneity is associated with increased agenda-setting success [18]. We identified two approaches related to problem definition. The first is a biomedical understanding of rehabilitation that focuses on expanding access to health services to improve functioning via a condition or disease of the body. The second is a rights-based, psychosocial approach to improving functioning via how an individual operates within society, inclusive of broader attitudinal and physical factors of the environment. The diversity in how rehabilitation is understood and framed leads to multiple ways of ‘making the case’ to policymakers and to the development of multiple solutions to advance different services or population needs.

In governance, rehabilitation actors face challenges organizing collectively. This is linked both to divergent problem definitions and the unclear boundaries of the issue. Consideration of the benefits and drawbacks of a diverse versus narrow coalition should be considered by rehabilitation advocates. Evidence emphasizes the importance of shared goals and incentives within a broad coalition, including health and non-health actors [27, 28, 41]. In rheumatic heart disease, emergency care, and surgical care, for example, narrowly focused coalitions of health experts have not sufficiently engaged other actors to advance their cause [24–26]. Conversely, a multisectoral coalition of rehabilitation actors – such as the diverse coalition of actors concerned with different forms of violence against children [23] – may face conflicting incentives when competing for scarce resources. This is particularly possible if different problems and solutions are being propagated by different sectoral actors.

The importance of a guiding institution during the agenda-setting phase is mixed, and coalitions do not require government leadership [16, 21, 23, 25, 27]. However, the role of ministries as guiding institutions may be particularly relevant for rehabilitation if identifying champions within government is a core means of elevating the agenda [18], or if formal coordination of a multisectoral approach is required [32].

Institutionalizing governance for rehabilitation within MOH has been advocated for. This could support prioritization by creating a clear line of accountability and empowering a guiding agency. However, we should not assume that centralization will automatically create stronger inter- and intra-agency coordination [65], particularly if rehabilitation leadership within the MOH lacks sufficient power or if increasing MOH resources results in animosity from other ministries. Further, strengthening MOH’s leadership in rehabilitation may marginalize the psychosocial and multisectoral framing of rehabilitation, losing support of a broader constituency. We were unable to identify comparative evidence on how rehabilitation has advanced in countries with different guiding institutions for governance, or more details on actor power and interests within rehabilitation communities (which will be context specific). This is an area for further research.

Finally, structural constraints influence governance and problem definition, particularly solution acceptability. Those advancing rehabilitation can explore how incremental health system reforms impact larger-scale prioritization. For example, pilots to advance service integration may provide a proof-of-concept and generate evidence. The emergence of effective solutions and their inclusion in international guidance was found to support prioritization of drowning [30], and the development of clear policy solutions supported maternal mortality prioritization [20]. However, challenges with scale-up or sustainability could contribute to existing fragmentation while further advancing the perception of intractability. Another important structural constraint is the historical exclusion of disability and rehabilitation from data systems. Evidence from drowning, maternal mortality, and pneumonia illustrates the importance of credible national indicators that show the extent of the problem [20, 30, 31]. We did not identify evidence from outside the health sector on how social service systems, and their relationship to the health system, may further influence structural factors. This is another area for future research.

These tensions point to a central question – can the rehabilitation field collectively overcome these challenges to advance a single agenda, or would smaller coalitions focused on specific conditions or services be more effective at making the case? Our findings challenge the idea that a cohesive rehabilitation community or coalition exists. Our goal is not to resolve these tensions, suggestions for which have been posed by others [46]. We instead provide a flexible framework for national stakeholders to guide further, context-specific research and action on these strategic considerations.

### Contributions of this framework to rehabilitation and the policy process literature

This study and the resulting policy framework make several contributions to the nascent evidence base on the prioritization of rehabilitation and broader policy process scholarship.

Specific to rehabilitation, this study complements a 2016 realist synthesis on good policy and governance for rehabilitation [33]. Our findings give further support to their emphasis on disaggregated disability statistics as an important advocacy input to quantify rehabilitation needs [33]. We build on their results to demonstrate how governance decisions create tradeoffs for prioritization. For example, institutionalizing rehabilitation programs within existing models of health care may support the sustainability of existing programs (a governance principle from the review) [33], but it could also reduce rehabilitation’s coalition outside the health sector, limiting multi-sectoral advocacy. Our framework can be utilized to analyze the country-specific implications of the rehabilitation governance principles on prioritization [33].

More broadly, the framework builds on existing policy theory and health issue prioritization frameworks in several ways. Existing policy theories are often developed from high-income country case studies and are non-health-sector specific. As a result, applying these theories to health issue prioritization in LMICs often excludes the influence of existing health system challenges in the agenda setting phase of the health policy process. But in this case, many of the rehabilitation-specific challenges we identify – lack of credible indicators, intractability, governance fragmentation – reflect longstanding structural factors of the health system. These directly contribute to the perception of intractability and fragmented governance arrangements, and in doing so, exert a path dependent influence on prioritization. For example, health information systems must be able to collect disability and rehabilitation-related indicators to generate credible indicators. Health workers must be trained to provide services before budgets can be allocated to provide those services. An abductive approach to analyzing our data surfaced these tensions, which may have remained “out of scope” if a pre-existing deductive policy framework for agenda setting was utilized.

The most prominent health-specific framework, Shiffman and Smith, is specific to global issue prioritization [16], while our framework is nationally-focused. Future research could apply the Shiffman and Smith framework to explore global-level prioritization of rehabilitation, and then utilize our framework to examine national-level prioritization in one or more countries.

### Limitations and their implications for application of the framework

Scholarship directs us to examine specific factors key to prioritization of an issue. In this case, the secondary data analyzed were primarily negative cases that identified challenges rather than successes. This allowed us to synthesize barriers to prioritization; however, we relied on informant recommendations and triangulation with theory to hypothesize enablers. Our literature review was a purposeful search to complement the interview data and should not be interpreted or appraised as a systematic review. It is possible that additional positive cases exist within the mental health or disability-specific literatures that were not identified through our purposeful, rehabilitation-focused search strategy.

While some of the theoretical literature we draw from has been deployed in LMIC health systems, others remain more focused on high-income country settings [65]. To overcome this, we reviewed empirical cases on how other health issues were prioritized in LMICs, which was further triangulated with interview data.

Taking a cross-country approach to developing the framework limited our ability to conduct a deeper analysis of context-specific considerations in a few ways. Extracted literature and policy theory focused largely on the national level; however, we recognize that sub-national prioritization is also critical. Our sample of KIs was largely at the international or national levels, with limited representation of local civil society organizations and no representation of patient organizations or advocacy groups. This was due to our inability to identify specific individuals to contact for virtual interviews in those stakeholder groups. Our literature search was also limited to publications in English, which may have reduced available literature from specific LMICs or regions. These limitations emphasize the need for cross-country validation, application and adaptation.

To facilitate national adaptation, we therefore kept the framework as components (rather than recommendations) and focused the components on the types of challenges common to national contexts rather than specific national challenges. A key consideration that did not emerge from our KII and literature data is the plausible influence of differing epidemiological profiles across countries. In South Africa, for example, 25% of rehabilitation-related needs are related to HIV-related conditions [90]. We would expect that this could influence all components of the framework, from how the need for rehabilitation is defined, to the actors involved in advocating for and providing services to patients, to the structure of the health and rehabilitation systems.

## Conclusion

We identify that problem definition, governance, and structural factors are all important to understanding prioritization of rehabilitation (or lack thereof) across national contexts. Our framework builds on the existing work of health policy scholars by emphasizing the importance of problem definition and governance and adds additional emphasis on the existing health system and historical legacies as structural factors that influence agenda-setting. The corresponding policy framework outlines a foundational approach to understanding these challenges and directs actors to a set of components core to advancing rehabilitation.

Application of this novel framework can support rehabilitation stakeholders in identifying the context-specific factors enabling or impeding the prioritization of rehabilitation in different national contexts. This is a critical first step for advancing rehabilitation on national policy agendas and ultimately, expanding equitable access to rehabilitative care in LMICs.

## Data Availability

All data generated or analysed during this study are included in this published article. All secondary data is included in the reference list, and quotes are included from the key informant interview data. Key informants were instructed that transcripts would not be shared.

## Abbreviations

CINAHL: Cumulated Index to Nursing and Allied Health Literature
COVID-19: SARS-CoV-2
CSO: Civil society organization
KII: Key informant interview
LMIC: Low- and middle-income country
MDG: Millennium development goals
MOH: Ministry of Health
NCD: Non-communicable disease
NGO: Non-governmental organization
PAHO: Pan American Health Organization
PHC: Primary health care
PWD: Persons with disabilities
STARS: Systematic Assessment of Rehabilitation Situation
UHC: Universal Health Coverage
USAID: United States Agency for International Development
WHO: World Health Organization
